# Nao-Xue-Shu Oral Liquid Improves Aphasia of Mixed Stroke

**DOI:** 10.1155/2015/709568

**Published:** 2015-10-19

**Authors:** Yuping Yan, Mingzhe Wang, Liang Zhang, Zhenwei Qiu, Wenfei Jiang, Men Xu, Weidong Pan, Xiangjun Chen

**Affiliations:** ^1^Shanghai Business School, Room 612, Administrative Building, No. 123, Fengpu Avenue, Fengxian District, Shanghai 201400, China; ^2^Department of Neurology, Shuguang Hospital Affiliated to Shanghai University of TCM, No. 528, Zhang-Heng Road, Pu-Dong New Area, Shanghai 201203, China; ^3^Department of Neurology, Shanghai Seventh Hospital, Shanghai University of Traditional Chinese Medicine, Shanghai 200137, China; ^4^Department of Emergency, Shuguang Hospital Affiliated to Shanghai University of TCM, No. 528, Zhang-Heng Road, Pu-Dong New Area, Shanghai 201203, China; ^5^Department of Neurology, Hua Shan Hospital Affiliated to Fu Dan University, No. 12, Wu Lu Mu Qi Zhong Road, Shanghai 200040, China

## Abstract

*Objective*. The objective is to observe whether the traditional Chinese medicine (TCM) *Nao-Xue-Shu* oral liquid improves aphasia of mixed stroke. *Methods*. A total of 102 patients with aphasia of mixed stroke were divided into two groups by a single blind random method. The patients treated by standard Western medicine plus *Nao-Xue-Shu* oral liquid (*n* = 58) were assigned to the treatment group while the remaining patients treated only by standard Western medicine (*n* = 58) constituted the control group. Changes in the Western Aphasia Battery (WAB), Modified Rankin Scale (mRS), National Institutes of Health Stroke Scale (NIHSS), and hemorheology parameters were assessed to evaluate the effects of the treatments. *Results*. Excluding the patients who dropped out, 54 patients in the treatment group and 51 patients in the control group were used to evaluate the effects. Significant and persistent improvements in the WAB score, specifically comprehension, repetition, naming, and calculating, were found in the treatment group when the effects were evaluated at the end of week 2 and week 4, respectively, compared with baseline. The naming and writing scores were also improved at the end of week 4 in this group. The comprehension and reading scores were improved at the end of week 4 in the control group compared with the baseline, but the improvements were smaller than those in the treatment group. The percentages of patients at the 0-1 range of mRS were increased at the end of week 2 and week 4 in both groups, but the improvements in the treatment group were much larger than those in the control group. Greater improvements in the NIHSS scores and the hemorheology parameters in the treatment group were also observed compared with the control group at the end of week 2 and week 4. *Conclusion*. *Nao-Xue-Shu* oral liquid formulation improved aphasia in mixed stroke patients and thus might be a potentially effective drug for treating stroke aphasia.

## 1. Introduction

Mixed stroke, also known as hemorrhagic infarction or infarction with hemorrhage, presents as a cerebral infarction combined with intracerebral hemorrhage on computed tomography (CT) brain scans [1, 2]. Current clinical cases of mixed stroke are caused by middle cerebral artery territory and lead to massive temporal infarction with hemorrhage. Mixed stroke patients with brain infarction and hemorrhage have mutual promoting and mutual transforming characteristics that often appear as epilepsy, dementia, aphasia, and other kinds of advanced neural function damage. Also, there is a higher proportion of patients with mixed apoplexy aphasia, including aphasia and dysarthria, or both kinds of symptoms coexisting in patients that result in communication difficulties, and loss of the ability to communicate socially has a serious impact on the patient's quality of life [3]. Western medicine treatment that consists of decreasing intracranial pressure and adjusting blood pressure and blood density and hemostatic measures can produce contradictory effects and, in other words, may lead to the development of ischemia and at the same time increase bleeding, and vice versa, causing contradiction to treat it [4]. In the theory of traditional Chinese medicine (TCM), one of the integrative medicines [5] has shown that* Nao-Xue-Shu* oral liquid may raise *Qi* and remove blood stasis, clear pathogenic “heat” and “cool” blood (make the abnormal activity of the blood quiet stop bleeding), and eliminate phlegm [6]. In Western medicine, the oral liquid can increase cerebral blood flow, improve microcirculation, prolong thrombus formation, bleeding, and clotting times, and inhibit platelet aggregation, so it can promote phagocytic function and accelerate the absorption of blood swollen in cerebral [6] and might be an effective prescription in the treatment of mixed stroke [7]. For these reasons, the aim of the present investigation was to evaluate whether* Nao-Xue-Shu* oral liquid can improve the aphasia of mixed stroke. We enrolled mixed stroke aphasia patients from the Department of Neurology of Shuguang Hospital affiliated to Shanghai University of Traditional Chinese Medicine and the Department of Neurology of Hua Shan Hospital affiliated to Fu Dan University based on whether or not they were taking* Nao-Xue-Shu* oral liquid in order to identify a reliable treatment for improving the prognosis of the patients.

## 2. Subjects and Methods

### 2.1. Subjects

A total of 116 mixed stroke patients with aphasia from our two hospitals were divided into a treatment group (treatment plus* Nao-Xue-Shu* oral liquid, *n* = 58) and a control group (treatment without* Nao-Xue-Shu* oral liquid, *n* = 58) in a single blind fashion. Inclusion criteria for the patients with aphasia of mixed stroke were (1) acute onset, neural function defect syndrome caused by a local brain blood circulation disorder, and duration of symptoms of at least 24 hours [8]; (2) diagnosis by CT and/or magnetic resonance imaging (MRI) of the brain clearly showing cerebral infarction accompanied by cerebral hemorrhage; (3) the patient in a conscious state and with the ability to speak and no comprehension difficulties, with or without dysarthria; and (4) the patient or their guardians providing signed informed consent. Exclusion criteria for those meeting the above inclusion criteria were (1) a patient with an existing consciousness disorder; (2) cerebral hemorrhage caused by another reason such as a tumor or brain trauma caused by cerebral infarction; (3) the existence of serious gastrointestinal bleeding, hemoptysis, or bloody urine; (4) the presence of other diseases caused by vascular dementia, frontotemporal dementia, Parkinson's disease (PD), Alzheimer's disease (AD), or a central nervous system disease such as a brain tumor, multiple sclerosis, encephalitis, epilepsy, normal pressure hydrocephalus (NPH), or other types of dementia; (5) alcohol and/or drug abuse or other known kinds of aphasia or dementia which prohibit the patient from cooperating with the examiner.

The mixed stroke patients with aphasia selected included 83 males and 33 females (age range, 39–87 y; mean ± SD, 64.28 ± 4.74 y), and time from onset to admission was 0.5~2.5 d (0.75 ± 1.08 d). There were 34 cases of left temporal infarction with hemorrhage, 23 cases of right temporal leaf infarction with hemorrhage, 14 cases of left putamen hemorrhage with right basal ganglia infarction, 12 cases of left putamen hemorrhage with right brain stem infarction, 11 cases of right basal ganglia infarction with hemorrhage in the left caudate nucleus, 9 cases of left cerebellar hemorrhage with right basal ganglia infarction, 8 cases of left thalamus hemorrhage with infarction in the right side of the basal ganglia area, and 5 cases of left basal ganglia infarction with right basal ganglia hemorrhage. No significant differences in gender, age, number of cases, duration, or types of diseases between the two groups were found, and the 2 groups were comparable (Table 1).

### 2.2. Treatment Methods

The control group underwent routine clinical treatments and measures according to the guidelines of Western medicine [9], including monitoring fluctuations in the electrocardiograph (ECG) and blood pressure. To control blood pressure and intracranial pressure,* mannitol* and/or* furosemidum* and* citicoline* were administered by intravenous infusion according to the patient's situation. The patients in the treatment group were treated using the same routine treatments as the control group and were also administered 10 mL of* Nao-Xue-Shu *oral liquid [6, 7] three times per day (Shandong* Wohua* Pharmaceutical Polytron Technologies Inc.), which consists of* Astragalus root*,* Hirudo, Acorus gramineus, Radix Achyranthis bidentatae, tree Peony bark, Rheum officinale,* and* Ligusticum wallichii* (batch numbers 5040504 and 5040708). The ratio formula of each herb or insect and the craftsmanship are protected by Chinese patent, but the effective elements could pass through the blood brain barrier to modify cerebral hemorrhage by study of cerebral hemorrhage model [10]. Patients who could not ingest the liquid orally were given it by nasal feeding. The patients in the treatment group took* Nao-Xue-Shu* oral liquid for 4 consecutive weeks. The clinical and laboratory parameters were measured before treatment (baseline), at the end of week 2, and at the end of week 4 to evaluate the effects of treatment in the two groups.

### 2.3. Assessments

(1) Western Aphasia Battery (WAB) [11] is the main outcome measure of aphasia. The examination not only detects fluctuations in aphasia but also assesses the use of visual spatial function, nonlinguistic intelligence abilities, spatial structure ability, ability to perform calculations, and other nonlinguistic function examinations. The WAB test has been used as a common tool in evaluating aphasia and is minimally influenced by race and cultural background in Western countries [12]. The six quotients developed by weighting WAB scores are as follows: comprehension, repetition, naming, reading, calculating, and writing, with the highest score being 100%.

(2) The Modified Rankin Scale (mRS) [13] is a simplification of the overall assessment of the patient's neurological function scale. The higher the score for neural function defect, the more serious the condition; 0 means no movement dysfunction and 6 means death. After 2 and 4 weeks of treatment, an increased percentage in the range of 0-1 of mRS will be used as the main determinant of improvement for movement dysfunction.

(3) The National Institutes of Health Stroke Scale (NIHSS) [14] as the reference index of curative effect include consciousness, gaze, facial paralysis, limb activities, and so on for a total of 11 scoring categories, with 0 points being normal. The higher the score of NIHSS, the more serious the neurologic deficit, NIHSS as a predictor of acute onset for stroke.

(4) Blood hemorheology as a reference index of the curative effect include whole blood viscosity low shear (WBVLS), whole blood viscosity high shear (WBVHS), plasma viscosity (PV), erythrocyte sedimentation rate equation *K* value (ESRE *K* value), fibrinogen, and erythrocyte aggregation index (EA index).

### 2.4. Statistics

SPSS17.0 software package was used for statistical analysis of the data. Data are presented as the mean and standard deviation (−*x* +* s*) or percentage (%). Repeated-measure ANOVA was conducted to test the differences among changes in outcomes at baseline and at the end of week 2 and week 4 for both groups. Differences at baseline between the treatment group and control group were analyzed. A *p* < 0.05 was considered to indicate a statistically significant difference.

## 3. Results

No significant differences in age, sex, educational level, handedness, aphasia type, baseline WAB score, mRS score, or NIHSS score, or blood parameters of hemorheology were observed between the treatment and control groups (Tables 1 and 2). After two weeks of treatment, there was one death in the treatment group due to severe lung infection, while three subjects died in the control group (one due to acute heart failure, one due to cerebral herniation, and one due to severe pulmonary infection). After 4 weeks of treatment, contact with three subjects in the treatment group was lost after they left the hospital. In the control group, two patients died and contact with two subjects was lost. Fifty-four patients in the treatment group and 51 patients in the control group were ultimately included in the statistical analyses.

The WAB scores in both groups at the end of week 2 and week 4 were better than their baseline scores. The scores for comprehension, repetition, reading, and calculation at the end of week 2 and week 4 in the treatment group were significantly improved compared with before treatment (baseline) (*p* < 0.05 or *p* < 0.01). These scores were much better at the end of week 4 in the treatment group than in the control group (*p* < 0.05). At the end of week 4, the WAB scores for naming and writing were better in the treatment group compared with baseline (*p* < 0.05), while only the comprehension and reading scores in the control group were significantly improved at the end of week 4. The levels of improvement at the end of week 4 were worse in the control group than in the treatment group (*p* < 0.05, Table 2).

**Figure 1:**
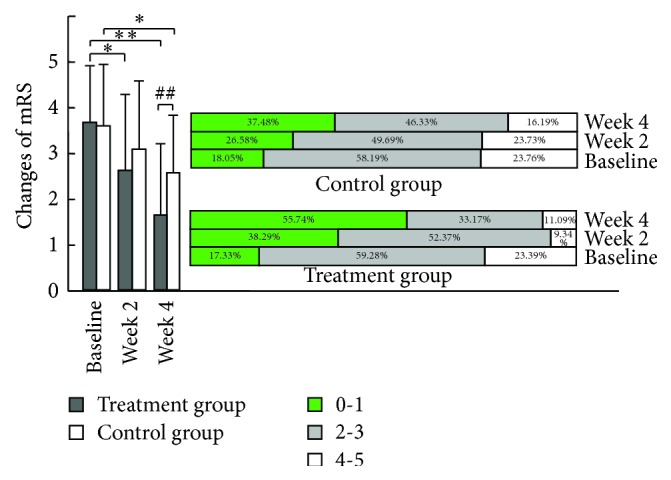
Changes in the modified Rankin score (mRS) between before and after the additional treatments in the treatment and control groups. Note: ^*∗*^ *p* < 0.05 and ^*∗∗*^ *p* < 0.01 compared with before for the same group; ^##^ *p* < 0.01 compared with control group at the same time.

The mRS score was significantly improved at the end of week 2 and week 4 in the treatment group (*p* < 0.05 and *p* < 0.01) compared with baseline, and the improvements were markedly better than those in the control group at the end of week 4 (*p* < 0.01). The mRS score only improved at the end of week 4 in the control group (*p* < 0.05) compared with baseline (Figure 1, left). The number of patients with an mRS score in the 0-1 range increased in both groups at the end of week 2 and week 4 (Figure 1, right), although the change was significantly greater in the treatment group.

**Figure 2:**
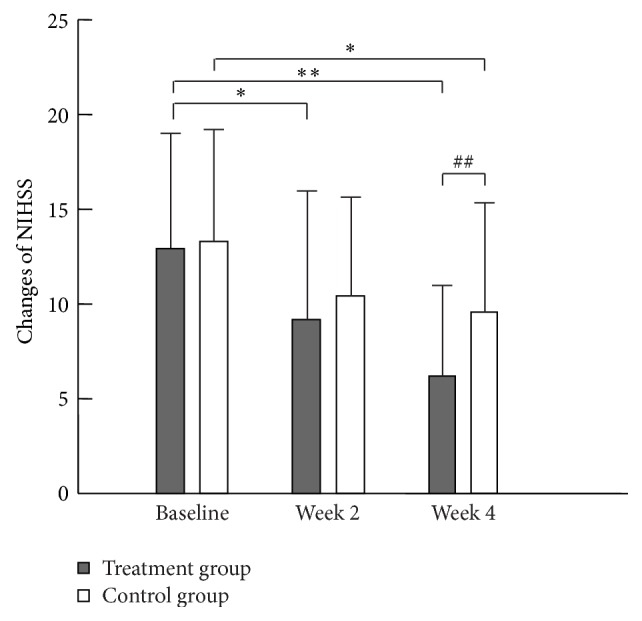
Changes in the National Institutes of Health Stroke Scale (NIHSS) scores between before and after the additional treatments in the treatment and control groups. Note: ^*∗*^ *p* < 0.05 and ^*∗∗*^ *p* < 0.01 compared with before for the same group; ^##^ *p* < 0.01 compared with control group at the same duration.

The NIHSS scores were improved at the end of week 2 and week 4 in both groups compared with their respective baselines, although the levels were markedly better in the treatment group than in the control group at the end of week 2 and week 4 (Figure 2).

The changes in most parameters in the blood hemorheology index in the treatment group at the end of week 4 were significantly different compared with baseline. The changes observed in all six parameters of the index in the treatment group were different compared with the control group at the end of week 4 (Table 3).

## 4. Discussion

There were more dropouts and deaths in the control group compared to the treatment group at the end of the study. Our results indicate that compared with baseline the treatment group (*Nao-Xue-Shu *oral liquid) had improved comprehension, repetition, reading, and calculating scores for aphasia parameters at the end of week 2 and the scores for these factors all had improved significantly at the end of week 4 compared with the control group and their baselines (Table 2). The treatment group exhibited improvements not only in the aphasia parameters but also in limb function, indicating that* Nao-Xue-Shu* oral liquid also can be used for treating patients with mixed stroke. After 4 weeks of treatment, the hemodynamic level of the treatment group improved compared with the control group, making it more close to the normal range (Table 3).

Mixed stroke is a common clinical cerebrovascular disease, and the patients experience acute onset and rapid progression. The cause of the disease is often an arterial lesion in the carotid artery system of the brain region, and the infarction area is large and often accompanied by damage to advanced brain function as the result of coma, aphasia, dementia, and epilepsy [15]. These have a serious impact on the quality of life and safety of the patient. Explaining the mechanism of action of* Nao-Xue-Shu* oral liquid in terms of traditional Chinese medicine (TCM) theory may be difficult to understand for most Western doctors. Mixed stroke in TCM is explained as “apoplexia” and an “attack on the viscera and bowels” [16], caused by a* Qi* deficiency, blood stasis, and phlegm. Due to the* Qi* deficiency, the blood stasis and phlegm obstruct the internal structure of blood vessel then intertwist each other, causing the blood stasis with phlegm to insert the vessel in the brain, leading to infarction. The abnormal blood causes intervessel high blood pressure, and forcing the blood stasis with phlegm out of the blood vessel may break the vessel, leading to hemorrhage [17, 18]. In TCM theory, if blood stasis is accompanied by phlegm, it can lead to a more significantly damaged lesion in the brain [19]. This is the mechanism that explains why mixed stroke patients often also have advanced neuronal damage, including aphasia, and the two pathological phenomenons of infarction and hemorrhage can be caused simultaneously. Physiologically, cleaning and powerful* Qi* (*Qing-yang Qi*) can supply energy to the brain to maintain its function and collect and modulate the blood and force it to circulate in correct way in brain blood vessels [17, 18]. If the circulation has been obstructed by the blood stasis with phlegm, the occlusion of blood vessel orifices will occur and the power of* Qi* will decrease;* Qing-yang Qi* is also like nutrition for the brain; if it cannot rise, it can lead to the brain lack of power to speak and understand the language and then can cause dysarthria and dysphagia. When treating this disease, we should consider three TCM pathogenic matters:* Qi*, blood stasis, and phlegm. First, we should eliminate* Qing-yang Qi*, which can modulate blood circulation and control or decrease bleeding. A*stragalus root *as a major component in* Nao-Xue-Shu* oral liquid can provide a stronger* Qing-yang Qi* [19]. The* Qi *also provides energy to raise the nutrient level in blood to the brain when treating the infarction and improves the aphasia. In TCM,* Qi *can improve circulation throughout the entire system and excrete metabolin. The other main component in the oral liquid is* hirudo, *a type of earthworm that has been used for more than one thousand years in China, which can rapidly eliminate blood stasis and treat the second pathogenic condition, that of blood stasis [20, 21], without side effect as bleeding. Other than these two components, the* Nao-Xue-Shu* oral liquid formulation contains 5 other TCM herbs that can help increase* Qi*, remove blood stasis and phlegm, and assist the body to excrete the pathogenic metabolites of blood stasis and phlegm. In fact,* Nao-Xue-Shu* oral liquid contains two famous prescriptions of TCM; one is* Bu-Yang-Huan-Wu decoction*, which originated in the Qing Dynasty (about 185 years ago) and has been used frequently to treat stroke in China and Asia [22, 23]. The other is* Da-Huang-Shu-Chong pill*, which comes from the very famous TCM text* Jin-Gui-Yao-Lue* (By Zhang Zhongjing, about 1700 years ago) and has been used to remove blood stasis from the body [24]. The combination of these 2 prescriptions is the most effective treatments in treating for mixed stroke with aphasia. Clinical pharmacological studies have confirmed that* Nao-Xue-Shu* oral liquid accelerates the absorption of hematoma in the brain of rats, reduces edema around the hematoma accelerating fibrinolysis and inhibiting thrombosis, increases cerebral blood flow, and improves brain blood and oxygen supply, thereby improving blood circulation and promoting the absorption of hematoma [25].

In this study on treating mixed stroke with aphasia, we believe the disease is caused by 3 pathogenetic mechanisms: a deficiency of* Qi, *blood stasis, and phlegm. The sample size of this study is relatively small and a single blind random method was used, so the treatment group might have experienced placebo effects and we therefore cannot draw any definite conclusion. In TCM theory, each Chinese medicine has its own function to modulate the body or deal with diseases, including treating brain problems, but doctors in China are still unable to demonstrate how the medicine passes through the blood brain barrier (BBB). Including TCM herbs [26, 27], many integrative medicines such as Ayurveda medicine [28], electric stimulation [29], and Tai Chi quan [30] cannot confirm that they influence the nerve system directly by Western medical technology, but they have been used in many countries for treating many diseases [5].* Nao-Xue-Shu* oral liquid contains a type of worm and this is another problem since, according to ethics, it is difficult to introduce such a treatment into foreign countries, although worms are frequently used in TCM treatments and TCM researchers in China have demonstrated they are harmless and safe. In order to validate the causes of the disease based on clinical data, large-scale, multicenter, double-blind randomized control studies will be needed to verify the effectiveness of* Nao-Xue-Shu* oral liquid in the treatment of mixed stroke aphasia.

## Figures and Tables

**Table 1:** Background characteristics of the patients of mixed stroke with aphasia.

Group	*n*	Gender		Age (y)	Educational level (*n*)			Handedness (*n*)		Aphasia type (*n*)		
		M	F		Primary	Middle	College or more	L	R	Motor	Receptive	Mixed
Treatment group	54	38	16	63.32 ± 5.1	13	24	17	5	49	23	19	12
Control group	51	37	14	64.6 ± 4.9	11	23	17	4	47	21	17	13

**Table 2:** Quantitative changes of Western aphasia battery (WAB) between before and after the additional treatments in the treatment and control groups.

	Comprehension	Repetition	Naming	Reading	Calculating	Writing
Treatment group						
Before	0.68 ± 0.22	0.53 ± 0.17	0.46 ± 0.31	0.57 ± 0.26	0.43 ± 0.37	0.62 ± 0.25
Week 2	0.76 ± 0.17^*∗*^	0.70 ± 0.32^*∗*^	0.52 ± 0.28	0.67 ± 0.25^*∗*^	0.56 ± 0.28^*∗*^	0.67 ± 0.21
Week 4	0.87 ± 0.12^*∗∗*#^	0.75 ± 0.21^*∗∗*#^	0.62 ± 0.24^*∗*#^	0.77 ± 0.18^*∗∗*#^	0.67 ± 0.22^*∗∗*#^	0.77 ± 0.12^*∗*#^
Control group						
Before	0.69 ± 0.23	0.54 ± 0.12	0.46 ± 0.25	0.56 ± 0.21	0.45 ± 0.29	0.63 ± 0.28
Week 2	0.70 ± 0.21	0.60 ± 0.19	0.49 ± 0.21	0.58 ± 0.24	0.48 ± 0.27	0.65 ± 0.25
Week 4	0.77 ± 0.19^*∗*^	0.63 ± 0.25	0.52 ± 0.17	0.65 ± 0.22^*∗*^	0.53 ± 0.33	0.68 ± 0.19

Note: ^*∗*^ *p* < 0.05 and ^*∗∗*^ *p* < 0.01 compared with before for the same group; ^#^ *p* < 0.05 compared with control group at the same time.

**Table 3:** Changes in hemorheology between before and after the additional treatments in the treatment and control groups.

	WBVLS (mPa·s)	WBVHS (mPa·s)	PV (mPa·s)	ESRE *K* value	Fibrinogen (g/L)	EA index
Treatment group (*n* = 54)						
Before	18.83 ± 4.36	3.82 ± 0.57	1.81 ± 0.52	68.27 ± 39.25	4.82 ± 1.25	4.72 ± 0.81
Week 2	17.96 ± 4.09	3.72 ± 0.35	1.68 ± 0.32	59.19 ± 41.62	4.19 ± 1.02	3.96 ± 0.63
Week 4	16.65 ± 3.74^*∗*#^	3.53 ± 0.32^*∗*#^	1.47 ± 0.44^*∗∗*#^	53.56 ± 40.69^*∗*#^	3.47 ± 0.72^*∗*#^	3.25 ± 0.52^*∗*#^
Control group (*n* = 51)						
Before	18.74 ± 5.05	3.82 ± 0.35	1.81 ± 0.37	67.82 ± 41.25	4.79 ± 1.46	4.70 ± 0.65
Week 2	18.38 ± 5.23	3.79 ± 0.62	1.76 ± 0.42	64.99 ± 39.02	4.02 ± 1.33	4.65 ± 0.92
Week 4	17.99 ± 4.59	3.77 ± 0.53	1.71 ± 0.38	63.47 ± 44.72	3.95 ± 1.49	3.97 ± 0.59

*Note*. WBVLS: whole blood viscosity low shear; WBVHS: whole blood viscosity high shear; PV: plasma viscosity; ESRE *K* value: erythrocyte sedimentation rate equation *K* value; and EA index: erythrocyte aggregation index. ^*∗*^ *p* < 0.05 and ^*∗∗*^ *p* < 0.01 compared with before for the same group; ^#^ *p* < 0.05 compared with control group at the same duration.
